# Improving heat resilience in fattening rabbits: nutritional strategies for mitigation via regulating blood physiology, inflammation and antioxidant pathways

**DOI:** 10.3389/fvets.2025.1677144

**Published:** 2025-09-29

**Authors:** Hadeel Kamil Khaleel, Sameh A. Abdelnour, Raha Osailan, Khaled H. El-Kholy, Ehab El-Haroun, Hamdy A. El-Nagar, Sara T. Mehilp, Ali A. El-Raghi, Mahmoud A. Hassan, Mahmoud Moustafa, Ibrahim T. El-Ratel

**Affiliations:** ^1^Department of Basic Science, College of Dentistry, Al-Iraqia University, Baghdad, Iraq; ^2^Department of Animal Production, Faculty of Agriculture, Zagazig University, Zagazig, Egypt; ^3^Biology Department, Faculty of Science, Taibah University, Yanbu, Saudi Arabia; ^4^Department of Animal, Poultry, and Fish Production, Faculty of Agriculture, Damietta University, Damietta, Egypt; ^5^Department of Integrative Agriculture, College of Agriculture and Veterinary Medicine, United Arab Emirates University, Al Ain, United Arab Emirates; ^6^Animal Production Research Institute, Agriculture Research Centre, Ministry of Agriculture, Giza, Egypt; ^7^Department of Biology, College of Science, King Khalid University, Abha, Saudi Arabia

**Keywords:** silver nanoparticles, rabbit, feed additives, inflammation, Apoposis

## Abstract

**Introduction:**

Global heat waves increasingly threaten food security, by reducing food supplies and negatively impacting livestock health. This research investigates nutritional interventions aimed at improving rabbit productivity and health, with a particular focus on mitigating the effects of heat stress (HS) conditions. Hence, this research investigates the potential of various feed additives to enhance growth, immunity, and overall health in environmentally stressed growing rabbits by reducing inflammatory and apoptotic signaling.

**Materials and method:**

To investigate the effects of various supplements on rabbits under natural heat stress, 150 rabbits were divided into five groups with 30 rabbits each. The groups were given a control diet or supplemented with 1 ml of silver nanoparticles (AgNPs), 0.2 g of chitosan, 100 mg of silymarin, or a combination of all three for 8 weeks. The Temperature-Humidity Index (THI) was calculated to be 30.04, indicating severe heat stress.

**Results:**

Results indicated that all feed supplements significantly improved live body weight and average daily gain (*p* < 0.05). The inclusion of dietary feed additives resulted in significant elevations in total protein (*p* < 0.01), albumin (*p* < 0.001), hemoglobin (Hb) (*p* < 0.01), and red blood cell (RBC) counts (*p* < 0.012) relative to the control diets. Conversely, dietary supplementation significantly attenuated serum concentrations of aspartate aminotransferase (AST; *p* < 0.001), triglycerides (TG; *p* < 0.001), total cholesterol (TC; *p* < 0.001), very-low-density lipoprotein (VLDL) (*p* < 0.001), urea (*p* = 0.04), and creatinine (*p* = 0.008) in comparison to the heat stress group. Administration of dietary supplements resulted in statistically significant elevations in total antioxidant capacity (*p* = 0.0032), glutathione (*p* = 0.002), catalase (*p* < 0.001), and glutathione peroxidase (*p* < 0.001), alongside a significant reduction in serum malondialdehyde (*p* = 0.0007), when compared to the control group. Similarly, these dietary interventions significantly enhanced overall immune ability, with increased IgM (*p* < 0.001), IgA (*p* = 0.0059), phagocytic activity (*p* < 0.001), and complement component 3 (C3) (*p* < 0.001). In addition, rabbits receiving dietary supplements displayed significantly reduced serum concentrations of pro-inflammatory cytokines, specifically tumor necrosis factor alpha (TNF-α, *p* = 0.0091), Interleukin 4 (IL-4, *p* = 0.0004), and interferon gamma (IFN-γ, *p* = 0.0211), compared with the stressed control group. Histopathological examination of liver tissues provided further evidence that all tested feed additives enhanced overall hepatic health.

**Conclusion:**

Therefore, incorporating mixture of natural molecules (1 ml AgNPs, 0.2 chitosan, and 100 mg silymarin) into the diet provides a sustainable and promising solution for rabbit production to combat heat stress associated with global climate change.

## 1 Introduction

The accelerating global climate change crisis poses substantial challenges to food security, rendering the livestock sector exceptionally vulnerable. Climate change can alter the spreading and prevalence of vector-borne diseases and parasites, affecting livestock health (1). Moreover, the escalating threat of rising temperatures and intensifying heatwaves imposes severe heat stress on livestock, leading to a cascade of detrimental effects. These include reduced feed intake, diminished milk yields, stunted growth, impaired reproductive capabilities, metabolic imbalances, and heightened mortality risks (2, 3). Heat stress (HS) is a most important environmental stressor that affects livestock production and can lead to economic losses. It occurs when animals are unable to dissipate heat effectively, resulting in a dangerous rise in center body temperature (1, 3).

Rabbit meat production is a growing global industry, driven by increasing consumer demand for its nutritional benefits (4). Valued for its high protein, vitamin, and mineral content, as well as its low saturated fat, rabbit meat is a healthy alternative to more traditional meats. This growing demand is contributing to the rapid expansion of the global rabbit production sector. Rabbit products are not only a dietary staple but are also being increasingly utilized in functional foods and pharmaceuticals (5). Despite this growth, the industry struggles with high environmental temperatures, which negatively impact its protein output (6). The absence of sweat glands renders rabbits highly susceptible to HS (7), a major challenge for production, particularly under hot environmental conditions (8, 9) HS can diminish growth efficiency and feed consumption [9], by compromising gut barrier function and microbiota (10), ultimately impairing nutrient availability and metabolism (11). Growth retardation may be attributed to inadequate feed supplies, while the inflammatory response and immune activation are metabolically demanding processes that require substantial nutrients and energy (11). Heat stress (HS) adversely impacts blood hematology, leading to anemia and cellular imbalances that can compromise immune function. This occurs through a reduction in the synthesis of immunoglobulins (IgM and IgG) and increasing the cortisol levels, essential for effective immune responses (12). The raised oxidative stress (OS) associated with HS can trigger lipid peroxidation and inflammation (13–15), ultimately compromising rabbit growth, productivity, and overall well-being (16). The inflammatory response is characterized by heightened activity and release of neutrophils, which are immune cells responsible for producing inflammatory mediators and reactive oxygen species. In response to heat stress (HS), this process includes the release of pro-inflammatory cytokines such as interleukin-4 (IL-4) and tumor necrosis factor-alpha (TNF-α) (15). To mitigate these effects in hot environments, several strategies have been investigated to augment the health of growing rabbits. These include genetic improvements, nutritional modifications, and optimized management practices.

Numerous feed supplements show promise in justifying the negative consequences of HS in rabbits and boosting their productivity (2, 17, 18). However, relying on antibiotics and chemical substances can lead to significant drawbacks, including the alarming rise of antibiotic resistance in pathogenic bacteria. Furthermore, some antibiotics can contaminate the environment and pose a threat to consumer well-being. Therefore, embracing natural substances as feed supplements presents a more environmentally sound and safer alternative for promoting growth, health, and reproductive efficiency (2, 17, 18).

Chitosan, a biocompatible biopolymer derived from chitin, exhibits significant therapeutic potential (19). It possesses several advantageous properties, including water solubility, low viscosity (20), biocompatibility, and non-toxicity (21). Furthermore, extensive research has demonstrated a broad spectrum of biological activities associated with chitosan counting antioxidant, anti-inflammatory, antibacterial, antifungal, antitumor, anti-obesity, antidiabetic, calcium absorption enhancement, and immune enhancement (22). Research indicates the potential of chitosan loaded with zinc oxide to improve heat resistance in broilers (23). Chitosan can combat heat stress by mitigating the damaging oxidative and inflammatory responses triggered by high temperatures (17). This action was demonstrated by regulating lipid metabolism disorders through enhancing the liver’s ability to promote the expression of *Nrf2, GPX1, PPARα,* and *CPT1*.

Phytotherapy, or herbal medicine, serves as an alternative to antibiotics. Silymarin, a flavonolignan extracted from milk thistle seeds (*Silybum marianum*) (24), is composed of flavonoids (silibinin, isosilibinin, silychristin, silidianin, and others). These flavonoids exhibit anti-fibrotic, antioxidants, anti-inflammatory, anti-lipid peroxidative, immunomodulatory, and hepatocytes-alleviating influences, and are used in the treatment of cirrhosis (25). Silymarin promotes ribosomal RNA synthesis in liver cells, encouraging protein production, and inhibits TNF-α production from isolated Kupffer cells and perfused rat livers due to its anti-inflammatory and anti-apoptotic effects (26, 27).

Silver nanoparticles (AgNPs) are gaining increasing attention for their potential applications in animal diets, primarily due to their antimicrobial properties (28). Silver has long been known for its ability to fight a broad spectrum of pathogens, involving viruses, bacteria, and molds. At the nanoscale, silver’s surface area and reactivity are significantly enhanced, making it even more effective (29, 30). AgNPs are recognized for their potent disinfectant and antimicrobial properties, reportedly exceeding the efficacy of other metallic nanoparticles. However, the potential for animal toxicity necessitates a cautious approach to their implementation in poultry production (29, 31). Previous research indicates that dietary supplementation with both nano-selenium and nano-silver can lead to their persistence and accumulation in organs such as the liver and spleen, potentially inducing toxicity upon prolonged exposure (32). AgNPs has been explored as a feed supplement, its high production cost has historically limited its competitiveness with antibiotics (33). Nevertheless, advancements in industrial synthesis and the regulatory ban on antibiotic growth promoters are renewing interest in nano-silver as a feed additive (34). Existing studies investigating the impact of Ag-NPs on animal health and performance have yielded conflicting results, demonstrating both detrimental and beneficial outcomes. Consequently, the present study aimed to comparatively evaluate the effects of selected natural molecules such as chitosan, silver nanoparticles (AgNPs), silymarin, and their combination on enhancing the thermotolerance of fattening rabbits. The investigation encompassed an assessment of their influence on growth parameters, blood biochemical profiles, hematological indices, digestive enzymes, immune competence, and the modulation of inflammatory and apoptotic cascades. The outcomes of this experiment may provide a feasible strategy for promoting sustainable rabbit production in the face of heat stress induced by climate change.

## 2 Materials and methods

### 2.1 Ethical record and resources of feed additives

Ethical approval for this trial and all rabbits handling procedures was performed and accepted by ZU-IACUC (Animal Care and Use Committee of the Laboratory Animal Center), University of Zagazig, Egypt (code: ZU-IACUC/2/F/367/2022). The study was handled in strict compliance with the appropriate rules and regulations of ZU-IACUC and the Animal Research Reporting of *In Vivo* Experiments (ARRIVE) guidelines. Chitosan and Silymarin were sourced from MISR Company for Food Additives, located in Badr City, Egypt. Silver oxide was provided by AB Chem Company, situated in Mansoura, Egypt.

### 2.2 Rabbits and experimental groups

This experiment was supervised through the summer season (July–August) at the Faculty of Agriculture, Egypt, to evaluate the consequences of various feed additives on rabbits. One hundred- and fifty-five-week-old weaned male New Zealand White rabbits (initial average weight: 665.35 ± 7.31 g) were sourced from University’s Rabbit Farm. These rabbits were randomly allocated to five treatment groups, with 30 rabbits per group (each two rabbits were considered as a replicate):

- Group 1 (Control): growing rabbits fed basal diet with no additives.
- The 2^nd^ treatment: growing rabbits fed basal diet fortified with silver nanoparticles (AgNPs) at 1 ml/kg, following previously established guidelines (32).
- The 3^rd^ treatment: growing rabbits fed basal diet fortified with 0.2 g of chitosan per kg of diet, based on (35).
- The 4^th^ treatment: growing rabbits fed basal diet fortified with 100 mg of silymarin per kg of diet (36).
- The 5^th^ treatment: growing rabbits fed the basal diet fortified with a combination of all three additives (1 ml AgNPs + 0.2 g chitosan + 100 mg silymarin per kg of diet).

All experimental diets, including the feed supplements, were prepared during the feed manufacturing process. From 5–13 weeks of age, rabbits were individually housed in 50 × 50 × 40 cm galvanized wire battery cages within a naturally ventilated facility. Consistent management and hygiene practices were maintained across all groups. Rabbits had *ad libitum* access to the basal pelleted diet and fresh tap water. The composition and chemical analysis of the basal diet, formulated according to nutritional guidelines for fattening rabbits (37), are detailed in Table 1.

**Table 1:** Ingredients and composition of basal diet of growing rabbits.

Ingredient	Basal diet (%)
Berseem hay	30.05
Barley grain	24.60
Wheat brain	21.50
Soybean meal	17.50
Molasses	3.00
Di-calcium phosphate	1.60
Limestone	0.95
DL-Methionine	0.15
Sodium chloride	0.30
Vitamins & Mineral Premix^(1)^	0.35
Total	100
Analyzed composition (%, on DM basis)	
Organic matter	91.42
Crude protein	17.36
Crude fiber	12.37
Ether extract	2.23
Nitrogen free extract	59.46
Ash	8.58

(1) Each 1 kg contains on Vitamin A (150, 000 UI), Vitamin E (100 mg), Vitamin B1(10 mg), Vitamin K3 (21mg), VitaminB2 (40 mg), Vitamin B6 (15mg), Vitamin B12 (0.1 mg), Pantothenic acid (100 mg) Niacin (200 mg), Biotin (0.5 mg), Folic acid (10 mg) and Choline chloride (5,000 mg). Each 1 kg contains on manganese (800 mg), zinc (600 mg), iron (300 mg), copper (40 mg), iodine (500 mg), selenium (100 mg), and cobalt (100 mg).

### 2.3 Meteorological attributes

In the rabbitry farm, an automatic thermo-hygrometer (Dostmann GmbH Wertheim, Germany) was used to daily record the relative humidity (RH, %) and air temperature. These readings allowed us to estimate the Temperature-Humidity Index (THI) via the subsequent formula:


$$\mathrm{THI}=\mathrm{dp}-[(0.31-0.31(\frac{\mathrm{RH}}{100}))x\;(\mathrm{dp}-14.4)]$$

Based on the reference (9), where dp represents the dry bulb temperature in Celsius (°C). The calculated THI values were then used to define levels of HS: a THI below 27.8 indicated no HS, 27.8 to 28.9 characterized moderate HS, 29.0 to 30.0 indicated severe HS, and values above 30.0 indicated very severe HS.

### 2.4 Growth, carcass attributes and chemical components

Growth parameters, involving live body weight (LBW), body weight gain (BWG), feed intake, and feed conversion ratio (FCR, expressed as grams of feed per gram of gain), were evaluated during the age intervals of 5–8, 8–11, and 5–11 weeks. The weights of rabbits were measured using a digital electronic balance with a sensitivity of +0.5 g.

At the end of the experiment (11 weeks), six rabbits from each treatment group were picked, subjected to a 12-h fast, weighed, and then humanely slaughtered according to Islamic guidelines (38). Following slaughter and complete bleeding, each carcass was partitioned into forequarters, loin, hindquarters, and toes. The weight of each portion was recorded and estimated as a percentage of the rabbit’s live weight before slaughter (39). The dressing ratio was calculated using the formula: (hot-dressed carcass weight/pre-slaughter weight) × 100 (40). Chemical analysis was performed on 100-gram dried meat samples to determine crude protein (CP), fat, and ash content. CP was measured using the Kjeldahl method with a Buchi analyzer and fat content was determined using the Soxhlet extraction method (Thermo Scientific, Warrington, UK). Ash content in the dietary samples was determined by incineration at 550 °C, as described by (40).

### 2.5 Blood hematology

In adherence to Islamic animal slaughter guidelines, anesthesia was not used according to (38, 41). During slaughter, we collected two blood samples from five rabbits per group. The first sample, drawn into sterile heparinized tubes, was immediately analyzed for hematological parameters using an automated hematology analyzer (Hospitex Hema Screen 18), following the methodology in (42). Key parameters measured included red blood cells (RBCs, 10^6^/mm^3^), white blood cells (WBCs, 10^6^/mm^3^), hemoglobin (Hb, g/dl), platelets, and packed cell volume (PCV, %). The second blood sample was allowed to clot, then centrifuged at 1200 g for 15 min to obtain serum, which was stored at −20 °C for subsequent biological analyses.

### 2.6 Blood biochemistry

To assess serum biochemical parameters in both stressed and treated rabbit groups, five samples were selected from each group for analysis. We measured total protein (TP, g/dl) and albumin (ALB, g/dl) concentrations using commercially available ELIKA Kits (Biodistogistic Company, Giza, Egypt). Globulin (GLB, g/dl) levels were calculated as the difference between TP and ALB. The albumin/globulin (ALB/GLB) ratio was subsequently determined. Serum levels of total cholesterol (TC, mg/dl), triglycerides (TG, mg/dl), high-density lipoprotein (Hdl, mg/dl), very low-density lipoprotein (VLDL, mg/dl), creatinine (mg/dl), and urea (mg/dl) were also determined. Additionally, we analyzed the activity of two liver enzymes, aspartate aminotransferase (AST, IU/L) and alanine aminotransferase (ALT, IU/L). These analyses were performed using a spectrophotometer and commercial kits from Bio-diagnostic Company (Giza, Egypt), adhering to the manufacturer’s instructions.

### 2.7 Digestive enzymes

Small intestinal digesta samples were collected from each of the three rabbits per group (n = 3) by manually expressing the contents from the entire intestinal tract. After weighing, each sample was diluted 1:10 in ice-cold phosphate-buffered saline (PBS, pH 7.0), then homogenized with a handheld glass homogenizer, and centrifuged at 2,000 g for 20 min at 4 °C. The resulting supernatant was separated and stored at 4 °C. Within 24 h of extraction, the activities of digestive enzymes (amylase, lipase, and protease) were determined using the methods described by (43).

### 2.8 Serum homeostasis

The total antioxidant ability (TAC) and the activities of key antioxidant enzymes, involving catalase (CAT), superoxide dismutase (SOD), and glutathione peroxidase (GSH-Px), were assessed in blood serum. Additionally, the levels of glutathione (GSH) and malondialdehyde (MDA) were determined. All analyses were performed using specific commercial kits (BioMérieux, France) conferring to the constructer’s commands.

### 2.9 TAC and MDA in liver, breast muscle and jejunum tissues

To determine total antioxidant capacity (TAC) and malondialdehyde (MDA) levels in liver, muscle, and jejunum tissues, we used distinct homogenization procedures. For hepatic samples, 500 mg were homogenized in phosphate-buffered saline, while separate 100 mg samples were homogenized in 1 ml of lysis buffer using a homogenizer. Both homogenates were then centrifuged at 3,000 rpm for 30 min. The resulting supernatants were collected and used to quantify TAC and MDA, following the manufacturer’s protocols (BioMérieux, Marcy-l’Étoile, France).

### 2.10 Immunity and prof-inflammatory cytokines

The concentrations of serum immunoglobulins (IgG, IgM, and IgA) in the experimental rabbits were determined using a method previously detailed by (44). Phagocytic activity was evaluated following the procedure outlined by (45). The inflammatory response in treated and control rabbits was assessed by measuring serum levels of TNF-α (tumor necrosis factor-alpha) (46), interferon-gamma (IFN-γ) (47), and interleukin-4 (IL-4) (48). These inflammatory biomarkers were quantified using ELISA kits purchased from MyBioSource (San Diego, CA, USA), and the assays were performed according to the manufacturer’s protocols. TNF-α (code: MBS2500169), IFN-γ (code: MBS705225), complement component 3 (C3, MBS701356), and IL-4 (code: MBS2018973) were detected in the ranges of 15.63–1,000 pg./ml for TNF-α, 62.5–4,000 pg./ml for IFN-γ, 27.3–7,000 ng/ml for C3 and 15.6–1,000 pg./ml for IL-4. The sensitivity values were 9.38 pg./ml for TNF-α, 30.4 pg./ml for IFN-γ, <27.3 ng/ml for C3, and 6.1 pg./ml for IL-4. Furthermore, serum lysosomal activity was evaluated using a 96-well microplate turbidity assay, as previously described by Aldal’in et al. (13). The levels of lysozyme activity (expressed as ng/ml) in serum was assessed through the uses of commercial ELISA kits, adhering to the protocols provided by the manufacturers.

### 2.11 Histopathological procedure

Utilizing previously described techniques (49), liver tissues from three rabbits per group were preserved in Bouin’s solution and prepared for histological analysis. This preparation involved sequential dehydration in ethanol (75 to 100%), clearing in xylol, and embedding in paraffin. The resulting 5-μm thick sections were stained with hematoxylin and eosin (H&E) and subsequently observed and documented using a Nikon Eclipse Ci-E microscope (Nikon, Japan) coupled with Motic Images Advanced 3.0 software.

### 2.12 Statistical analysis

The results were first documented using Microsoft Office Excel, version 16. The Shapiro–Wilk test (50) was employed to assess the normality of the data. Subsequently, various parameters, including growth, carcass and feed utilization indices, chemical composition, hematology, oxidative stress, immune features, and inflammatory responses, were analyzed using the MIXED procedure (PROC MIXED) in SAS software.

The subsequent statistical model was applied:


Yij=μ+Ti+eij,

Where Yᵢⱼ represents the observed value for a given treatment, μ is the overall mean, Tᵢ is the fixed effect of the treatment, and eᵢⱼ is the random error term accompanying with each observation.

Multiple comparisons of means were achieved utilizing Duncan’s multiple range test (51). Statistical significance was established at *p* < 0.05.

## 3 Results

### 3.1 AgNPs characterization and climatic aspects

**Figure 1:**
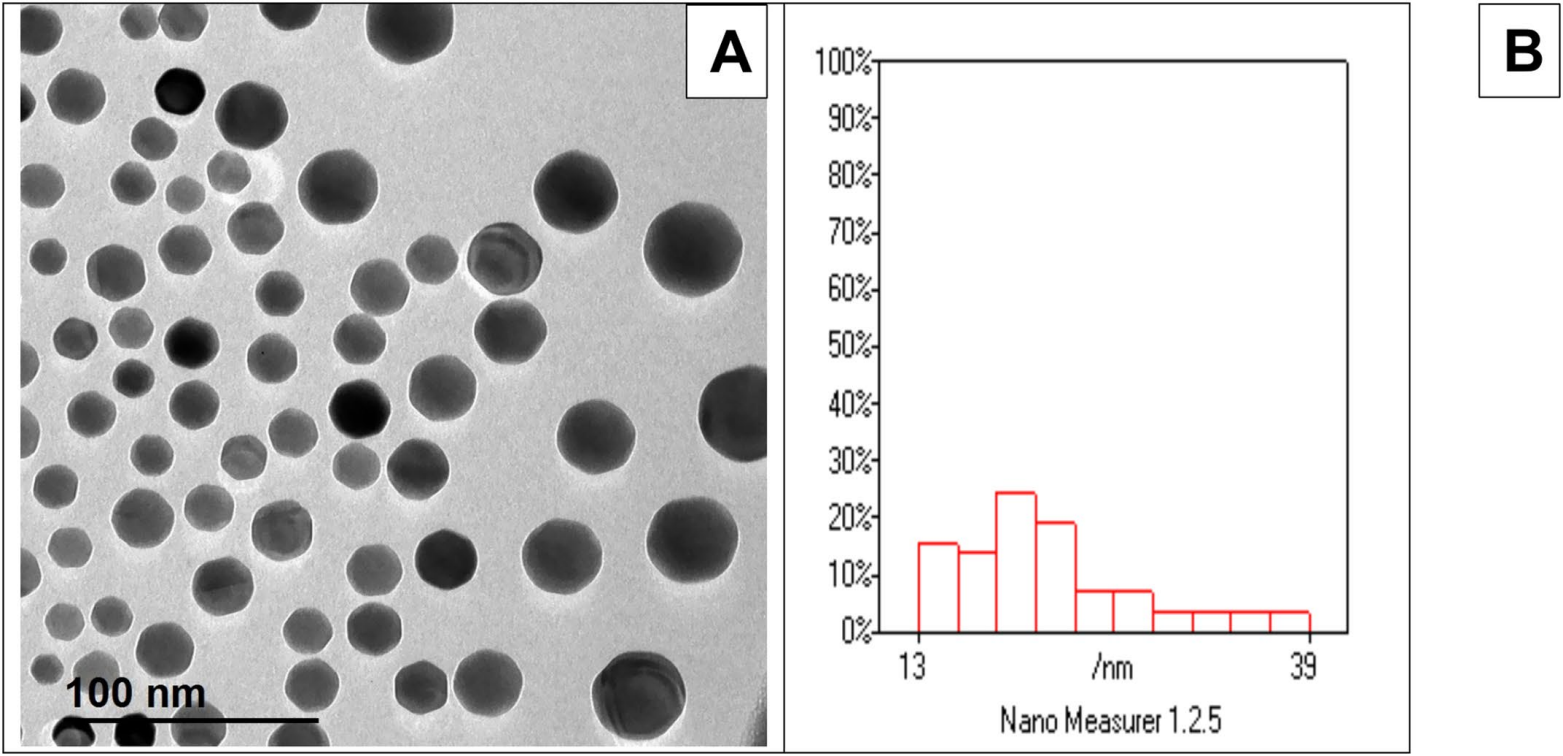
**(A)** TEM analysis revealed that the Ag-NPs exhibit a predominantly spherical morphology. **(B)** corresponding histogram indicated a broad particle size distribution, with most particles clustering between 13 and 39 nm.

**Figure 2:**
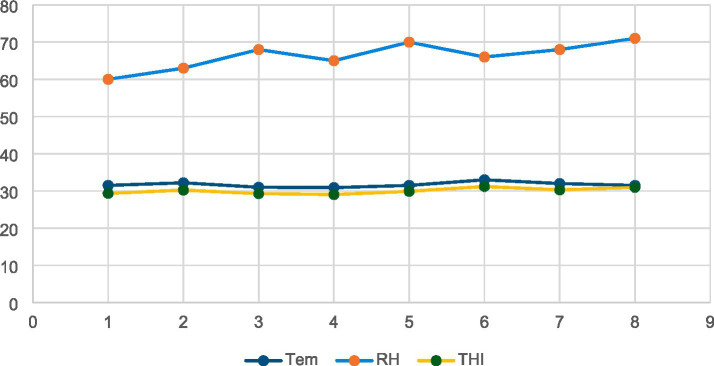
The THI, relative humidity (RH) and ambient temperature (Tem) during the study period.

Figure 1A shows the morphology of Ag-NPs by TEM showing nearly spherical particles. Figure 1B Histogram shows that most particles cluster around 13 to 39 nm, with a good distribution. Based on the collected data, the ambient temperature, relative humidity, and THI were 31.7 °C, 66.38%, and 30.04, respectively, indicating that the rabbits were exposed to natural environmental heat stress (Figure 2).

### 3.2 Growth attributes

Table 2 demonstrates a significant improvement in LBW across all supplemented additives compared to the untreated group at both 8 and 11 weeks of age (*p* < 0.001). Stressed rabbits fed with various additives showed significantly higher ADG than the control group during the 5–8-week period and overall (*p* < 0.001). In contrast, there was no substantial variation in ADG between the groups during the 8–11-week period (*p* > 0.05). Neither feed intake nor FCR was significantly affected by any of the supplemented feed additives in rabbits kept under HS conditions (*p* > 0.05).

**Table 2:** Growth performance, and feed utilization of heat-stressed growing rabbits as affected by dietary silver nanoparticles (Ag-NPs), chitosan, silymarin and their mixture.

Item	Experimental groups^1^					SEM	*p* value
	Control	Ag-NPs	Chitosan	Silymarin	Mixture		
Live body weight (g)							
5 wks	666.58	665.30	666.40	665.35	664.85	7.3110	0.9998
8 wks	1237.47^b^	1295.95^a^	1311.00^a^	1310.45^a^	1318.95^a^	12.470	<0.0001
11 wks	1863.21^b^	1929.15^a^	1944.40^a^	1945.45^a^	1962.50^a^	18.837	0.0033
Average daily gain (g)							
5–8 wks	27.19^b^	30.03^a^	30.70^a^	30.72^a^	31.15^a^	0.441	<0.0001
8–11 wks	29.79	30.15	30.16	30.24	30.65	0.755	0.9595
Overall	28.49^b^	30.09^a^	30.43^a^	30.48^a^	30.90^a^	0.429	0.0015
Feed intake (g/day) at:							
5–8 wks	91.21	94.00	95.25	96.80	95.100	3.8123	0.8851
8–11 wks	134.47	133.35	138.90	136.20	139.15	3.470	`0.6967
Overall 5–11 wks	112.84	113.68	117.08	116.50	117.13	2.535	0.6432
Feed conversion ratio (g feed/g gain)							
5–8 wks	3.38	3.14	3.09	3.16	3.06	0.137	0.5647
8–11 wks	4.52	4.43	4.93	4.52	4.55	7.456	0.4287
Overall, 5–11 wks	3.97	3.78	3.99	3.83	3.79	0.149	0.7921

^1^Rabbits fed a basal diet (control) or the basal diet supplemented with 1 ml AgNPs, 0.2 g chitosan, 100 mg silymarin, or their mixture for 8 weeks under environmental heat stress. Weeks (wks). Results are presented as mean ± pooled standard error (pooled SE). Statistical significance was considered at *p* < 0.05.

### 3.3 Digestive enzymes

**Figure 3:**
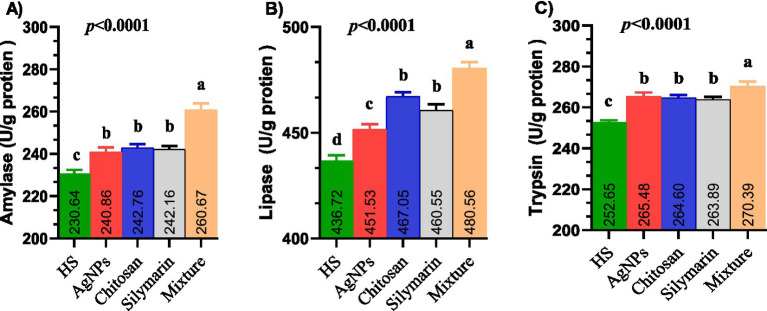
**(A–C)** The digestive enzymes activities such as amylase **(A)**, lipase **(B)** and trypsin **(C)** of heat-stressed growing rabbits as affected by dietary silver nanoparticle, chitosan, silymarin and their mixture. Rabbits fed a basal diet (control) or the basal diet supplemented with 1 ml AgNPs, 0.2 g chitosan, 100 mg silymarin, or their mixture for 8 weeks under environmental heat stress.

Supplementation of the diet with all examined additives led to a significant improvement in the activity of digestive enzymes (Figure 3), specifically amylase (Figure 3A), lipase (Figure 3B), and trypsin (Figure 3C), with the combined additive group showing the highest values (*p* < 0.01). Individual feed additives had similar effects on amylase and trypsin activity, while lipase activity was significantly higher in the chitosan and silymarin groups compared to the AgNPs group.

### 3.4 Carcass traits, and chemical composition

The levels of crude protein, fat, and ash did not show significant difference among the various feed additive groups compared to the stressed control group (*p* > 0.05, Table 3). Furthermore, the dietary inclusion of feed supplements did not have a significant impact on carcass characteristics (*p* > 0.05).

**Table 3:** Carcass traits, and chemical composition of heat-stressed growing rabbits as affected by dietary silver nanoparticle (Ag-NPs), chitosan, silymarin and their mixture.

Item	Experimental groups^1^					SEM	*p* value
	Control	Ag-NPs	Chitosan	Silymarin	Mixture		
Carcass traits (%)							
Carcass	55.55	54.92	56.77	56.28	56.78	1.222	0.7781
Dressing	58.46	59.65	58.47	59.49	60.45	2.131	0.9571
Fore part	15.1	14.76	15.07	14.96	15.42	1.522	0.8170
Mid part	17.12	18.6	16.37	17.99	17.38	0.963	0.8874
Hind part	26.24	26.29	27.03	26.54	27.65	0.788	0.4582
Internal organs (%)							
Liver	3.15	3.24	3.26	3.28	3.44	0.1452	0.1326
Kidney	0.62	0.60	0.61	0.64	0.62	0.7523	0.8542
Lung	0.60	0.61	0.60	0.62	0.63	0.8524.	0.7516
Spleen	0.08	0.09	0.12	0.10	0.11	0.035	0.5423
Chemical composition (%)							
Crude protein	17.56	17.83	18.24	18.35	18.59	0.516	0.6357
Fat	3.33	3.29	3.32	3.31	3.30	0.109	0.9994
Ash	1.94	1.91	1.93	1.88	1.95	0.127	0.9954

^1^Rabbits fed a basal diet (control) or the basal diet supplemented with 1 ml AgNPs, 0.2 g chitosan, 100 mg silymarin, or their mixture for 8 weeks under environmental heat stress. Results are presented as mean ± pooled standard error (pooled SE). Statistical significance was considered at *p* < 0.05.

### 3.5 Blood hematology and serum metabolites

For hemoglobin (Hb) and RBCs counts, AgNPs yielded similar results (Table 4). However, dietary supplement with chitosan, silymarin, and the combination of additives led to significantly higher Hb levels linked to the basal diet (*p* < 0.01). The combination group exhibited the highest RBC count, with the chitosan and silymarin groups showing RBCs counts that were not extensively unique from the combination group (*p* > 0.05). WBCs, Platelets and hematocrit did not affect by the treatment (*p* > 0.05). Feeding stressed rabbits diets supplemented with various additives led to increased total protein (TP) and albumin levels, with the combination group displaying the maximum values (*p* < 0.05). Conversely, serum globulin, A/G ratio, HDL, and ALT levels remained unaffected by these dietary supplements (*p* > 0.05). However, the inclusion of these supplements significantly reduced serum AST, TG, TC, VLDL, urea, and creatinine in stressed rabbits (*p* < 0.05).

**Table 4:** Blood hematology and serum metabolites of heat-stressed growing rabbits as affected by dietary silver nanoparticles (AgNPs), chitosan, silymarin and their mixture.

Item^1^	Experimental groups^2^					SEM	*p* value
	Control	AgNPs	Chitosan	Silymarin	Mixture		
Hematological parameters							
Hb (g/dl)	10.50^b^	10.43^b^	11.68^a^	11.70^a^	12.03^a^	0.359	0.0111
RBCs (10^6^/mm^3^)	5.65^c^	5.50^bc^	6.08^ab^	6.06^ab^	6.22^a^	0.149	0.0112
WBCs (10^3^/mm^3^)	6.36	6.30	6.15	6.12	6.01	0.152	0.5108
Platelets (10^3^/mm^3^)	261.71	263.54	279.27	280.62	290.98	9.838	0.2175
Hematocrit (%)	34.71	33.49	29.09	29.38	28.82	1.679	0.0673
Biochemicals parameters							
TP (g/dl)	6.28^c^	6.36^b^	6.49^b^	6.52^b^	6.93^a^	0.018	0.0050
Albumin (AL, g/dl)	3.36^b^	3.34^b^	3.70^a^	3.71^a^	3.96^a^	0.098	0.0010
Globulin (GL, g/dl)	2.92	3.02	2.78	2.82	2.97	0.176	0.8567
AL/GL ratio	1.16	1.14	1.35	1.38	1.33	0.112	0.4216
TG (mg/dl)	90.10^a^	85.44^b^	74.88^bc^	72.76^bc^	65.70^c^	2.737	<0.0001
TC (mg/dl)	112.24^a^	105.82^b^	100.24^b^	101.45^b^	90.78^c^	0.452	<0.0001
HDL	46.42	47.49	48.67	49.61	50.84	3.166	0.8742
VLDL	18.02^a^	17.09^a^	14.98^b^	14.55^bc^	13.14^b^	0.547	<0.0001
Creatinine (mg/dl)	1.29^a^	1.20^ab^	1.02^bc^	1.07^c^	0.93^c^	0.052	0.0008
Urea (mg/dl)	38.87^a^	32.49^b^	31.27^b^	31.94^b^	28.50^b^	2.228	0.0400
AST (IU)	37.48^a^	30.47^b^	25.90^c^	25.57^c^	23.85^c^	1.446	<0.0001
ALT(IU)	49.77	44.50	42.24	42.95	41.33	2.524	0.1776

Results are presented as mean ± pooled standard error (pooled SE). Statistical significance was considered at *p* < 0.05.

1 Hemoglobin (Hb), red blood cells (RBCs), White blood cells (WBCs), total protein (TP), triglycerides (TG), total cholesterol (TC), high density lipoprotein (HDL), very low-density lipoprotein, aspartate aminotransferase (AST) and alanine aminotransferase.

2 Rabbits fed a basal diet (control) or the basal diet supplemented with 1 ml AgNPs, 0.2 g chitosan, 100 mg silymarin, or their mixture for 8 weeks under environmental heat stress.

### 3.6 Oxidative related biomarkers

Dietary feed additive supplementation had no significant impact on SOD levels (*p* = 0.1073, Table 5). However, various supplemented additives significantly enhanced the levels of TAC (*p* = 0.0032), GSH (*p* < 0.001), CAT (*p* < 0.001), and GPX (*p* = 0.002) in rabbits. The combination group displayed the highest TAC, CAT, and GPX levels (*p* < 0.05). Chitosan and silymarin supplementation in stressed rabbits resulted in similar TAC levels to other treatments (*p* > 0.05). The AgNPs group showed intermediate TAC, GSH, and MDA values (*p* < 0.05). Furthermore, all feed supplements effectively reduced MDA levels, with the combination group exhibiting the most substantial reduction (*p* < 0.05).

**Table 5:** Antioxidants capacity in blood serum of heat-stressed growing rabbits as affected by dietary silver nanoparticles (Ag-NPs), chitosan, silymarin and their mixture.

Item^1^	Experimental groups^2^					SEM	*p* value
	Control	Ag-NPs	Chitosan	Silymarin	Mixture		
TAC (ng/ml)	0.35^c^	0.40^b^	0.48^ab^	0.50^ab^	0.55^a^	0.034	0.0032
GSH (U/ml)	9.45^c^	11.73^b^	14.08^a^	14.29^a^	14.75^a^	0.576	<0.0001
SOD (U/ml)	0.330	0.370	0.400	0.420	0.470	0.036	0.1073
CAT(U/ml)	32.26^c^	39.49^b^	45.29^b^	44.20^b^	48.77^a^	1.002	<0.0001
GSH-Px (U/ml)	4.46^c^	4.88^b^	5.24^b^	5.29^b^	5.59^a^	0.141	0.0002
MDA (nmol/ml)	15.89^a^	12.30^b^	10.67^c^	10.74^c^	9.12^d^	0.937	0.0007

Results are presented as mean ± pooled standard error (pooled SE). Statistical significance was considered at *p* < 0.05.

1 Total antioxidant capacity (TAC), glutathione (GSH), superoxide dismutase (SOD), catalase (CAT), and glutathione peroxidase (GSH-Px), and malondialdehyde.

2 Rabbits fed a basal diet (control) or the basal diet supplemented with 1 ml AgNPs, 0.2 g chitosan, 100 mg silymarin, or their mixture for 8 weeks under environmental heat stress.

### 3.7 Tissues oxidative markers

**Figure 4:**
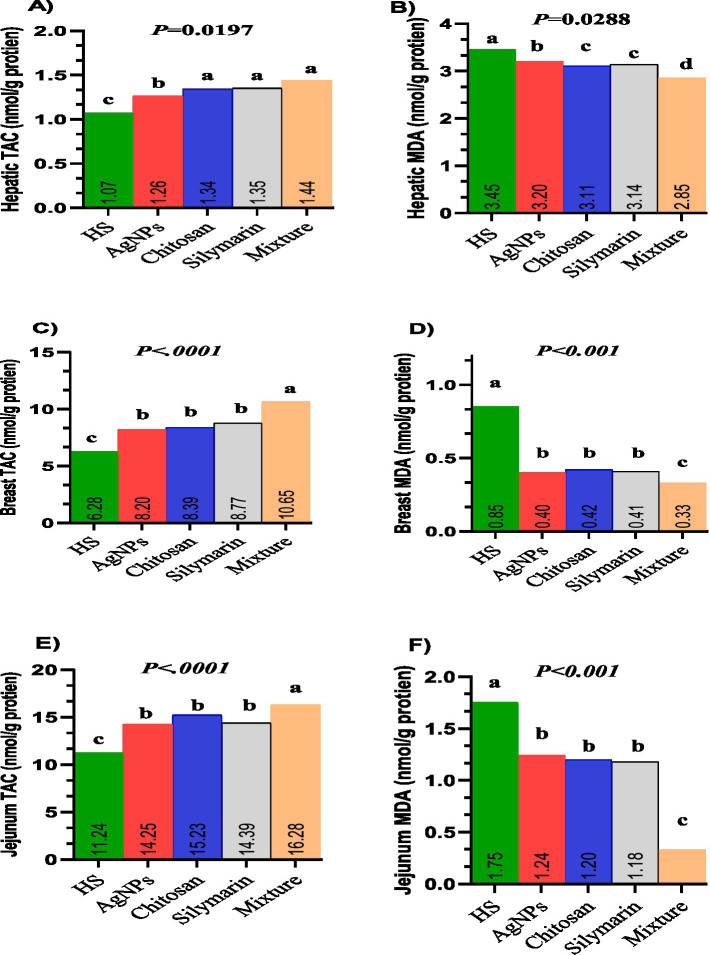
**(A–F)** Tissues oxidative markers of heat-stressed growing rabbits as affected by dietary silver nanoparticle, Chitosan, Silymarin and their mixture. TAC **(A)** and MDA **(B)** in liver tissue. TAC **(C)** and MDA **(D)** in breast muscle tissues. TAC **(E)** and MDA **(F)** in jejunum tissues.

Figures 4A–F illustrates the effects of different feed additives on total antioxidant capacity (TAC) in the liver (Figure 4A), muscle (Figure 4C), and jejunum (Figure 4E), as well as on malondialdehyde (MDA) levels in the liver (Figure 4B), muscle (Figure 4D), and jejunum (Figure 4F) of growing rabbits. Supplementing the diets of stressed rabbits with various molecules significantly improved TAC in liver tissues (*p* = 0.019) and significantly reduced lipid peroxidation (*p* = 0.0288), with the lowest MDA levels observed in the combination group. Rabbits fed diets supplemented with feed additives exhibited the highest TAC in both breast muscle and jejunum tissues, with the combination group displaying the greatest TAC levels (*p* < 0.01). Conversely, stressed rabbits fed treated diets showed significantly lower MDA levels, with the lowest MDA values observed in the combination group (*p* < 0.05).

### 3.8 Immune function and pro-inflammatory cytokines

Dietary fortification of stressed rabbits with select natural molecules resulted in significant improvements in IgM (*p* < 0.001), IgA (*p* = 0.0059), and phagocytic activity (*p* < 0.001) relative to the untreated group (Table 6). The combination feed additive led to the highest observed levels of IgM, phagocytic activity, and IgA (*p* < 0.05). Dietary supplementation with various molecules in rabbits led to higher complement component 3 (C3) levels compared to stressed rabbits (*p* = 0.0087). The AgNPs group exhibited intermediate C3 values (*p* < 0.05). All supplemented molecules resulted in lower levels of tumor necrosis factor-alpha (TNF-α) (*p* = 0.0091), interleukin-4 (IL-4) (*p* = 0.0004), and interferon-gamma (IFN-γ) (*p* = 0.0211) compared to the stressed control group. Serum TNF-α and IL-4 levels did not show significant differences among rabbits fed AgNPs, chitosan, or silymarin; however, these groups differed significantly from other treated groups (*p* < 0.05). Serum IFN-γ levels were similar in the chitosan and silymarin groups, displaying the lowest values, while the AgNPs and combination groups showed a similar trend for IFN-γ levels. Serum lysosome activity wasn’t affected by the treatment (*p* = 0.1406).

**Table 6:** Immune function and pro-inflammatory cytokines of heat-stressed growing rabbits as affected by dietary silver nanoparticles (Ag-NPs), chitosan, silymarin and their mixture.

Item^1^	Experimental groups^2^					SEM	*p* value
	Control	Ag-NPs	Chitosan	Silymarin	Mixture		
Immune function							
IgG (mg/dl)	12.80	13.11	13.44	13.50	13.74	0.127	0.3221
IgM (mg/dl)	0.52^c^	0.58^b^	0.63^b^	0.62^b^	0.69^a^	0.113	<0.0001
IgA (mg/dl)	0.68^c^	0.72^b^	0.75^b^	0.77^b^	0.80^a^	0.023	0.0059
Phagocytic (%)	27.28^c^	34.21^b^	34.44^b^	36.24^b^	44.28^a^	0.247	<0.001
Inflammatory cytokines							
C3 (ng/ml)	18.69^c^	26.13^b^	27.69^a^	29.42^a^	31.67^a^	1.935	0.0087
TNF-α (pg/ml)	109.76^a^	93.58^b^	85.46^b^	85.24^b^	80.15^c^	4.889	0.0091
IL-4 (pg/ml)	118.07^a^	110.9^b^	95.95^b^	96.06^b^	89.87^c^	4.295	0.0004
IFN-γ (pg/ml)	90.13^a^	81.31^b^	77.72^c^	76.77^c^	72.53^b^	2.914	0.0211
Lysozyme (pg/ml)	1.92	2.10	2.20	2.19	2.28	0.099	0.1406

Results are presented as mean ± pooled standard error (pooled SE). Statistical significance was considered at *p* < 0.05.

1 Tumor necrosis factor-alpha (TNF-α), interferon-gamma (IFN-γ), interleukin-4 (IL-4), and complement component 3 (C3).

2 Rabbits fed a basal diet (control) or the basal diet supplemented with 1 ml AgNPs, 0.2 g chitosan, 100 mg silymarin, or their mixture for 8 weeks under environmental heat stress.

### 3.9 Histological findings

**Figure 5:**
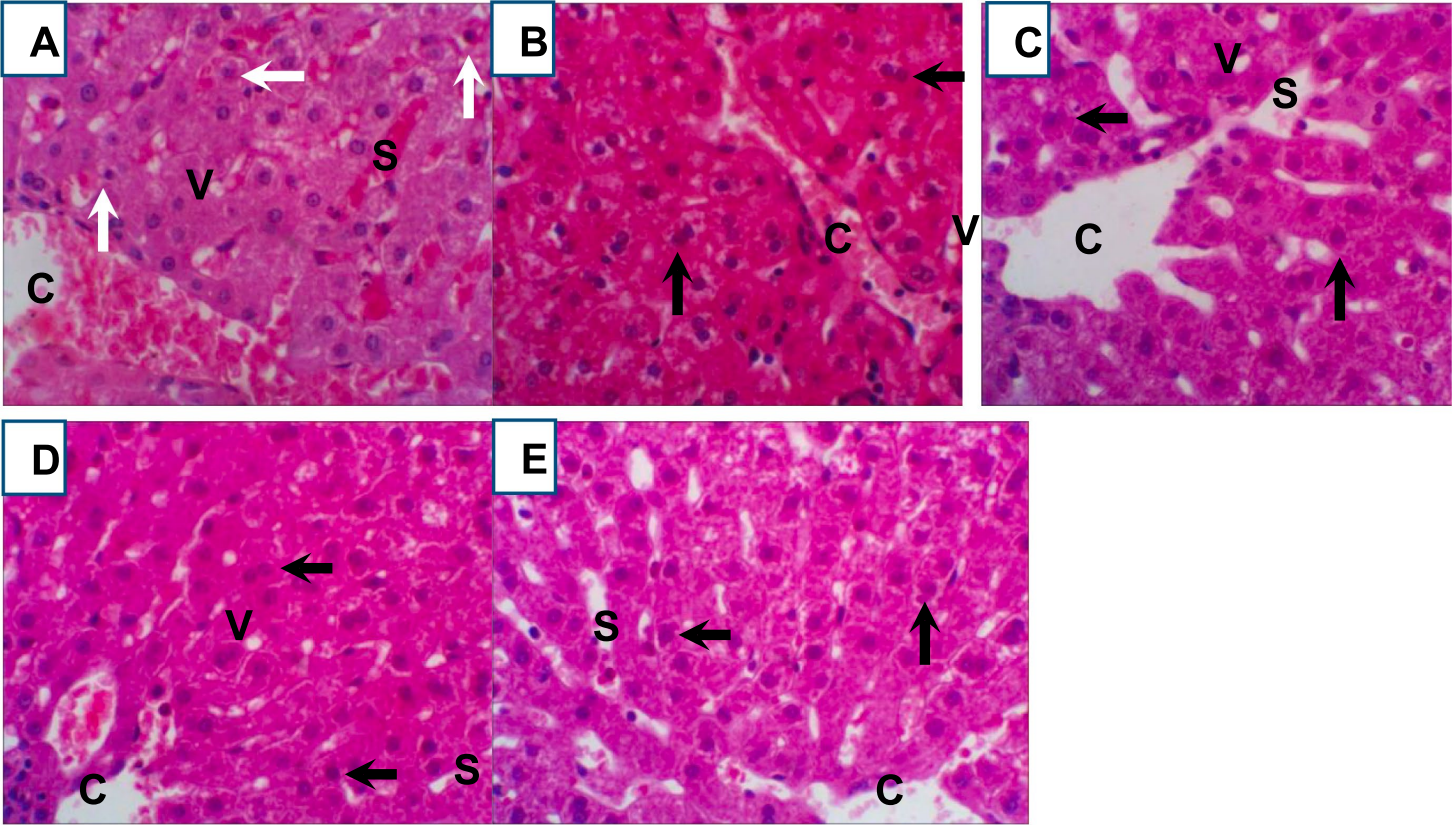
**(A–E)** Representative photomicrographs (400x magnification) of hematoxylin and eosin (H&E) stained rabbit liver tissue sections from control and treated groups: **(A)** heat stress control, **(B)** Ag-NPs treated, **(C)** chitosan treated, **(D)** silymarin treated, and **(E)** combination treated. Key histological features are indicated: central vein (CV), dilated sinusoid (S), hepatocyte degeneration (white arrow), normal hepatocytes (black arrow), and vacuolated hepatocytes.

Histopathological analysis of liver tissues from heat-stressed growing rabbits in the control group (Figure 5A) revealed significant alterations, including congested and dilated central veins, and marked hepatic cell degeneration. Furthermore, hepatocytes exhibited degeneration characterized by prominent cytoplasmic vacuoles (v), sinusoidal dilatations (S), and evidence of blood infiltration in the heat stress control group. In contrast, the Ag-NPs treated demonstrated a moderate amelioration of hepatic architecture (Figure 5B). Liver sections from this group showed a more organized hepatic parenchyma with hepatic lobules radiating around central veins, normal sinusoids, and hepatocytes with minimal cytoplasmic vacuolation. Notably, the liver sections from rabbits in the chitosan, silymarin, and combination treatment groups (Figures 5C–E) displayed near-normal hepatocyte structures, including the parenchymal cells and central veins, indicating a reduction in the inflammatory cell infiltration induced by severe heat stress.

## 4 Discussion

Global climate change continues to be a significant concern with broad effects on all organisms across the planet, exerting a substantial influence on the rabbit industry. Given the decreased productivity often observed and the inferior thermotolerance of rabbits to HS, scientists have implemented various nutritional interventions aimed at enhancing productive and health parameters for sustainable production in tropical environments. This study found that the natural HS conditions were determined using the THI, with a value of 30.04 indicating severe HS according to the model by (9). Studying natural environmental HS is particularly relevant compared to simulated HS in animals due to the complex interactions among various environmental factors present in natural settings. Therefore, rabbits were maintained under natural HS conditions and fed diets supplemented with various natural molecules to assess their comparative efficacy, including a mixture of these molecules.

Overall, the results obtained showed significant improvements in growth and health aspects across all supplemented diets, with the mixture group exhibiting the most pronounced effects. However, AgNPs appeared to produce a more limited improvement, while silymarin and chitosan were more effective in enhancing physiological parameters, blood homeostasis, and reducing inflammatory and apoptotic cascades in growing rabbits. Growth indices and carcass traits are important in the rabbit industry. AgNPs are well-known for their antimicrobial activity (34, 52, 53), while chitosan is a widely found polymer in many insects, and silymarin has exhibited hepatoprotective properties.

Some studies support our findings regarding the enhanced growth observed with the dietary inclusion of AgNPs in rabbits (32). In contrast, a study showed that 100 ppm of zinc oxide nanoparticles (Zn-CNPs) did not impact the growth performance of growing rabbits. However, the study did find a significant improvement in nutrient digestibility (DM, OM, CP, EE, and NFE), as well as increased hot carcass, dressing, and spleen weights (17). Contrary to some reports, and aligning with our findings, dietary chitosan supplementation significantly improved growth metrics and hot carcass wight, liver in rabbits (15). The observed growth improvement can be attributed to chitosan’s capacity to increase the solubility of digesta within the gastrointestinal system, which in turn facilitates nutrient digestibility and enhances absorption. Several studies have demonstrated that chitosan, when combined with materials like berberine nanoparticle (54) or gentamicin conjugate (55) can significantly enhance the growth performance of heat-stressed rabbits. The beneficial effects of chitosan are thought to be driven by its positive charge, which allows it to bind with the negatively charged nucleic acids of pathogenic bacteria like *E. coli* and their associated toxins (56). This binding has a dual impact: it can directly disrupt bacterial synthesis, and by neutralizing toxins, it may also upregulate occludin protein expression, ultimately strengthening the tight intestinal junctions.

Moreover, dietary supplementation with chitosan has been shown to enhance the activity of key digestive enzymes such as protease, lipase, and amylase, which likely contribute to the efficient breakdown of dietary components into absorbable nutrients within the intestinal lumen. Silymarin is widely recognized for its hepatoprotective effects against toxins such as aflatoxin (27) and CCl₄ (57). Also, the growth-promoting effects observed are likely a result of silymarin’s dual action as a hepatoprotective and immunostimulatory agent. Bioactive compounds such as flavonoids and phenolics, commonly found in plant extracts, are believed to enhance feed digestibility and nutrient bioavailability, ultimately improving feed utilization and protein synthesis. Moreover, the presence of bioactive phytochemicals, including those in silymarin, may contribute to improved growth by increasing feed intake, efficiency, and protein retention (58). Silymarin’s potential to stimulate protein synthesis through enzymatic pathways (59), as well as its demonstrated influence on growth hormone gene expression related to muscle development further supports these growth benefits. AgNPs have been widely used for disinfection and antimicrobial activity, while their effects as a growth promoter and anti-heat stress in rabbits agent are scarce (32). This study found minor improvements in some growth indices due to AgNPs, while its combination with other molecules significantly improved the growth of stressed rabbits. A study by (53) suggested that silver’s effectiveness might be more pronounced under stress conditions associated with increased pathogenic bacteria (29). These hypotheses could partially explain the variability in prior findings. Furthermore, the discrepancies in results may stem from differences in animal species, nanoparticle size, synthesis techniques, administration dosage, and delivery methods. In congruence with our findings, AgNPs did not significantly impact LBW in rabbits and broilers (29). However, other studies have revealed significant improvements in growth following AgNPs administration (32). Our findings indicate that dietary Ag-NPs enhanced digestive enzyme activity in rabbits. This observation is consistent with a report by (32) which noted increased protease, amylase, and lipase levels in Ag-NP-treated Nile tilapia. Although the precise mechanism through which silver influences digestive enzymes is not yet understood, its resemblance to established roles of zinc and copper in promoting intestinal villi growth and pancreatic activity suggests a potential, similar pathway.

Blood is classified as a connective tissue characterized by a fluid matrix, the plasma, within which various cellular components are suspended, namely erythrocytes, leukocytes, and thrombocytes. Its primary function is to connect the body’s organs and cells, facilitating the maintenance of a stable cellular environment. This is achieved through continuous circulation, delivering essential nutrients to all tissues and removing metabolic waste products. Dietary chitosan supplementation at 500–1000 mg/kg improved hematocrit (Hct), but did not affect other hematological parameters (15). Silymarin also improved the HCT and RBCs in stressed growing rabbits (57). Moreover, a study by (17) reported that Zn-CNPs significantly increased protein, HDL, and albumin levels, while also significantly decreasing (*p* < 0.05) glucose, total cholesterol, and LDL concentrations. The ability of chitosan to enhance hepatic antioxidant and anti-inflammatory capacity contributes to the alleviation of heat-stress-induced lipid metabolism disorders (60, 61). Silymarin administration led to improvements in blood protein levels and a reduction in liver enzyme and kidney function markers, bringing them within physiological ranges (57). Additionally, the combined application of all tested feed additives supported the sustained health of liver and kidney tissues under environmental stress conditions. These results were supported by other authors (57, 62). The hypocholesterolemic effect was also reported, manifesting as decreased serum cholesterol, LDL, and triglycerides, along with eminent HDL cholesterol in rabbits (57). Silymarin is highly binds and lipophilic to plasma membrane constituents, enhancing plasma membrane asset and dipping membrane rupture and disintegration (63). Supplementation with silymarin mitigated and prevented the adverse effects of Salinomycin sodium in growing rabbits (26). This was evidenced by its protective effects through enhancing the liver and kidney histological structure. Dietary AgNPs in rabbits resulted in improved liver and kidney function (32). Based on our study, the administered doses of AgNPs were shown to be safe for rabbits, as evidenced by their normal liver and kidney function. The elevated levels of total protein and globulin may be associated with increased immunoglobulin fractions. The reduced levels of AST and creatinine suggest a positive impact of Ag-NPs on health, potentially mediated by their enzymatic stimulator, antimicrobial, anti-inflammatory, antioxidant, and immunostimulatory properties. These findings partially align with the work of (31), who reported a significant increase in TP in broiler diets supplemented with 2.5 mg Ag-NPs/kg, while albumin, globulin, and urea showed non-significant increases. Conversely, the results of (30) indicated that significant increases in blood globulin and AST in broilers supplemented with Ag-NPs (2–10 ppm).

Heat stress in growing rabbits triggers oxidative damage, evidenced by increased body temperature, elevated levels of ROS, and MDA, consequently weakening the cellular defense system (12, 13, 64). This is supported by previous research linking HS to metabolic alterations and oxidative damage in skeletal muscle due to ROS overproduction. Dietary chitosan oligosaccharides supplementation demonstrated a protective effect (15, 61). Our study showed that chitosan improved TAC and reduced MDA in various tissues, also enhancing TAC, CAT, GSH, and GSH-Px activity. This is consistent with previous research on chitosan showing MDA reduction and SOD/GSH-Px increase under chronic heat stress, alleviation of H_2_O_2_-induced ROS/MDA increases (11), and reduced doxorubicin-induced ROS production (65). In stressed mice, 200 mg of chitosan significantly enhanced GSH-Px and GSH levels, while reducing the concentration of IL-1β (61). In stressed rats, oral administration of 500 mg of chitosan significantly improved the levels of GSH-Px, CAT, and TAC, while reducing MDA levels (19). Silymarin was also found to enhance cellular defense due to its antioxidant capacity (27). The observed reduction in ROS and MDA, coupled with increased CAT activity in the treated group, suggests that dietary chitosan can partially alleviate AHS-induced oxidative damage. However, the lack of a significant difference in CAT activity between the treated groups indicates that the protective mechanisms of chitosan may not primarily involve direct enhancement of antioxidant enzyme activity.

Dietary fortification of stressed animals with natural substances possessing immunomodulatory actions may enhance their thermotolerance and improve survival rates. IgA, IgM, and phagocytic activity were significantly improved by the addition of feed additives in stressed rabbits. Zn-CNPs improved IgG, IgM in rabbit (17). Several studies have demonstrated that chitosan can reduce the levels of pathogenic bacteria, including *E. coli* and *Salmonella* spp., in the cecum of rabbits (22, 55, 56). Polysaccharides like chitosan are known immunomodulators affecting antigen-presenting cells and T cells. The existing study found an increase in serum IgM, and C3 levels with higher dietary chitosan, consistent with previous research in piglets and oligochitosan-fed subjects (17, 61). Chitosan particles may enhance innate immunity by promoting phagocytosis. Oligochitosan’s prebiotic properties in hens have been suggested, possibly through bacterial interaction as an antigen. Our findings align with prior research indicating that Ag-NPs can enhance immunity through various mechanisms (32). These include stimulating lymphocyte production, inducing heat shock protein synthesis without inflammation, and potentially improving cellular oxygenation. Ag-NPs’ antimicrobial properties may indirectly influence immunity via gut microbiota modulation (66, 67). Direct immunomodulation by Ag-NPs involves altering cytokine expression (28) and promoting lymphoid organ development, possibly through enhanced anabolic activity and oxygen supply (52). Notably, Ag-NPs have also shown promise in enhancing disease resistance (52), although their impact on immunoglobulin levels appears variable across studies (31).

Chitosan has shown promise in alleviating the damaging consequences of HS, particularly through its positive impact on intestinal function. In broilers, chitosan successfully modulated gut health to counteract HS (68). Similarly, studies on growing rabbits have demonstrated that dietary chitosan (500–1,000 mg/kg) substantially enhanced villus length and width, enhanced crypt depth, boosted goblet cell numbers, and reduced muscular layer thickness (15). Chitosan has the potential to reduce inflammation in stressed rats by modulating the expression of cytokines, specifically increasing IL-10 and decreasing TNF-α (19). A study by (61) reported that chitosan improved the health status of stressed mice via regulating the inflammatory responses. The addition of dietary feed additives significantly reduced the serum levels of C3, TNF-α, IL-4, and IFN-γ. These reductions could reflect the potential anti-inflammatory activity of all tested feed supplements. Notably, recent studies underscore the therapeutic potential of chitosan in inflammatory conditions, where they have been shown to suppress the invention of key inflammatory mediators like prostaglandin E2, nitric oxide, and pro-inflammatory cytokines (69). Silymarin’s anti-inflammatory effects in several *in vivo* acute inflammation models (70). Silymarin effectively mitigated carrageenan-induced paw edema when administered orally to rats. Its anti-inflammatory effect was also demonstrated against xylene-induced mouse ear inflammation, where topical application of silymarin showed greater efficacy (44.52% inhibition) than indomethacin (35.96%) (70). Notably, intraperitoneal administration of silymarin yielded even more potent results. Silymarin also inhibited carrageenan-induced leukocyte migration in mice dose-dependently, reducing leukocyte accumulation and neutrophil migration. While ineffective against phospholipase A2, *in vitro* assays revealed silymarin’s ability to inhibit lipooxygenase and cyclooxygenase (71).

Histological analysis typically reveals hepatocyte destruction associated with HS. However, in this study, histological examination of hepatic tissues demonstrated that all supplemented feed additives conferred hepatoprotective effects against HS-induced liver injury. In heat-stressed control rabbits, liver tissues exhibited congested and dilated central veins, hepatic cell degeneration, hepatocyte degeneration with vacuoles, sinusoidal dilation, and blood infiltration (72). The Ag-NPs group showed moderate improvement with more normal hepatic structure. Chitosan, silymarin, and their mixture groups displayed nearly normal hepatocyte structures and reduced inflammatory cells compared to the heat-stressed control. Consistent with prior studies highlighting the hepatoprotective properties of silymarin (27, 57, 62) and chitosan (21, 22, 54), these results support these findings. In contrast (73), previously reported the therapeutic potential of AgNPs in the context of cadmium-induced hepatotoxicity, a different form of liver injury.

## 5 Conclusion

The findings of the present study suggest that the inclusion of specific natural compounds (1 ml AgNPs, 0.2 chitosan, and 100 mg silymarin) synergistically enhances heat resistance in growing rabbits. This was demonstrated by improved growth, overall health, and a reduction in lipid peroxidation, which can be attributed to the modulation of post-metabolic functions and digestive enzyme activity, as well as the enhancement of cellular antioxidative mechanisms. The supplements also mitigated inflammatory markers (C3, TNF-α, IL-4, IFN-γ) and improved immune function (IgM, IgA, phagocytic activity) in stressed animals. Consequently, this dietary strategy represents a promising method to enhance animal thermotolerance in the face of climate change. Additional molecular studies are needed to further validate these findings.

## Data Availability

The raw data supporting the conclusions of this article will be made available by the authors, without undue reservation.
